# Effects of Topical Emu Oil on Burn Wounds in the Skin of Balb/c Mice

**DOI:** 10.1155/2016/6419216

**Published:** 2016-03-16

**Authors:** Mohammad Afshar, Reza Ghaderi, Mahmoud Zardast, Parvin Delshad

**Affiliations:** ^1^Department of Anatomy, Faculty of Medicine, Birjand University of Medical Sciences, Moallem Street, Birjand 9717735338, Iran; ^2^Medical Toxicology Research Center, Mashhad University of Medical Sciences, Mashhad, Iran; ^3^Department of Dermatology, Faculty of Medicine, Birjand University of Medical Sciences, Moallem Street, Birjand 9717735338, Iran; ^4^Faculty of Medicine, Birjand University of Medical Sciences, Moallem Street, Birjand 9717735338, Iran

## Abstract

The goal of this study was to determine the effect of topical Emu oil on the healing of burn wounds and hair follicle restoration in superficial II-degree burns in the skin of Balb/c mice. Thirty-two male Balb/c mice with burns on the back of the neck were divided into two groups: The Emu oil group received topical Emu oil twice daily, whereas the control was left untreated. Skin biopsies were obtained on days 4, 7, 10, and 14 of the experiment. Then the specimens were viewed with Olympus SZX research microscope. The Emu oil treated burns were found to heal more slowly and inflammation lasted longer in this group. The number of hair follicles in the margins of the wounds increased through time in the Emu oil group compared to the control group. Also, the hair follicles in the Emu oil group were in several layers and seemed to be more active and mature. Moreover, Emu oil had a positive effect on fibrogenesis and synthesis of collagen. The findings indicate that although Emu oil delays the healing process, it has a positive effect on wound healing and it increases the number of hair follicles in the margins of the wound.

## 1. Introduction

Burn injuries, today, are one of the most common reasons for illness and death in the world. The final goal of the usual treatment is to facilitate the healing of skin, which is the first body's barrier against microbes and dehydration [1]. The treatment of burn wounds has always been one of the biggest concerns of mankind. Treating wounds of this kind—due to different reasons—can cause great difficulties in the practice of medicine [2]. Various kinds of synthetic ointments and creams have been used to treat burn wounds. Each of which has its own limitations and side-effects. With a modern outlook on complementary medicine during the past few years, the application of different oils and natural products has been proposed for treating burn wounds.

Having conducted an experiment on rats, Hosseinimehr et al. claimed that Aloe Vera cream, in comparison to silver ointment, causes a smaller scar and speeds the reepithelialization process in rats after being scolded with boiled water [3]. Tarameshloo et al. have also reported positive effects of Aloe Vera gel on wound healing [4]. Similar studies have been conducted on sea buckthorn [5], saffron [6], banana tree leaves [7], and honey [8].

Emu oil, derived from the Emu (*Dromaius novaehollandiae*), is also used in traditional medicine for treating wounds and has been reported to have anti-inflammatory effects [9]. The Emu (*Dromaius novaehollandiae*) is a large bird whose oil was used by Australian aboriginals and the early white settlers to facilitate wound healing and alleviate pain from various musculoskeletal disorders [10]. Emu oil consists of long chained triglyceride esters, such as Oleic acid and Linoleic acid and also saturated fatty acids like palmitic acid and stearic acid [11]. Also, different amounts of compositions, such as carotenoids, flavons, polyfenols, and tocoferols, are present in the nontriglyceride part of Emu oil, which can result in favorable antioxidant effects [11].

There are some studies on the healing effects of the Emu oil especially on its anti-inflammatory qualities [11, 12]. One study in CD-1 mice found that the auricular swelling induced by applying 50 *μ*L of croton oil was significantly reduced 6 hrs after the application of Emu oil, when compared to the control and the porcine oil groups [12]. A second study, using both female outbred Wistar and Dark Agouti rats with adjuvant-induced polyarthritis, revealed significant reductions in paw swelling (up to 84%) and arthritis score (up to 70%) upon exposure to Emu oil [13]. There have been a few studies carried out on the healing properties of Emu oil on the skin [14]. There is some evidence showing that Emu oil delays the healing of burn wounds [14]. On the other hand, Gong et al., in a study on 144 Wistar male rats, showed that the topical application of Emu oil in second-degree burn wounds has a favorable, anti-inflammatory effect and improves wound healing by inhibiting the secondary inflammation process [15].

Considering the controversy that has been mentioned above and due to few comparative studies on the healing effects of Emu oil on burn wounds, this study was conducted to test Emu oil healing effects on second-degree burn wounds on the skin of Balb/c mice.

## 2. Materials and Methods

32 male Balb/c mice (obtained from Pastor Laboratory, Tehran, Iran), with a mean body weight of 25 ± 5 gr, were used in this experiment. All animal experiments were performed with the approval of the Institutional Animal Care and Use Committee at Birjand University of Medical Sciences. They were well housed in separate cages, having easy access to food and water. The light/dark cycle was 12 : 12. The environment temperature was 22-23 degrees Celsius and the humidity was 45–50%.

Pure Emu oil (Kalaya) was used in this study. Kalaya Emu oil is produced under stringently controlled conditions in order to guarantee the highest quality product. Testing has shown that this oil has primary compositions such as Oleic acid, Linoleic acid (omega 6 EFA), and alpha-linolenic acid (omega 3 EFA). The amount of omega 3 EFA of this product is four times higher than industry standard.

After shaving the back of their necks, the mice were anesthetized with 70 mg/kg intraperitoneal ketamine. Subsequently, they were randomly assigned to two groups: Emu oil group and control group. After that, a superficial burn wound (deep second degree) was made on the shaven area by the use of a handmade device. The device was a nail-like, metal piece, the flat top of which was 1 cm in diameter, heated for 3 minutes on an alcohol lamp and exposed to the skin for 10 seconds. Then, 0.5 cc of Emu oil was applied twice daily (8 am and 8 pm) on the burn wounds of the Emu oil group and rubbed for 30 seconds, whereas the control group was left untreated. On days 4, 7, 10, and 14 of the experiment, 4 samples from each group were randomly selected and euthanized to obtain skin biopsies.

For euthanasia, the mice were put in a closed transparent jar filled with high doses of ether to stop their cardiac rate and respiration. Subsequently, skin biopsies were obtained and fixed in formalin 10%. Having passaged the samples through the common histological process (dehydration with alcohol, clearing with Xylene), the samples were embedded in paraffin. Later, each sample was trimmed through serial section into a hundred 5-micron thick slices with the help of the Leica RM2235 Rotary Microtome. Ten slides were randomly selected from each sample and stained with Hematoxylin-Eosin and Trichrome-Mallory dyes.

To gather the data and assess the variables (number of hair follicles, inflammatory cell density, fibroblast density, and granulation tissue formation), the glass slides were observed by two pathologists and were later photographed by the Olympus SZX research microscope equipped with a camera. The magnification scales were 40x and 400x. The collected data were then analyzed with the SPSS software package using the Friedman, Wilcoxon, Mann-Whitney, and Fisher exact tests (*P* < 0.05).

## 3. Results

### 3.1. Microscopic Features of Wounds on Day 4

**Figure 1:**
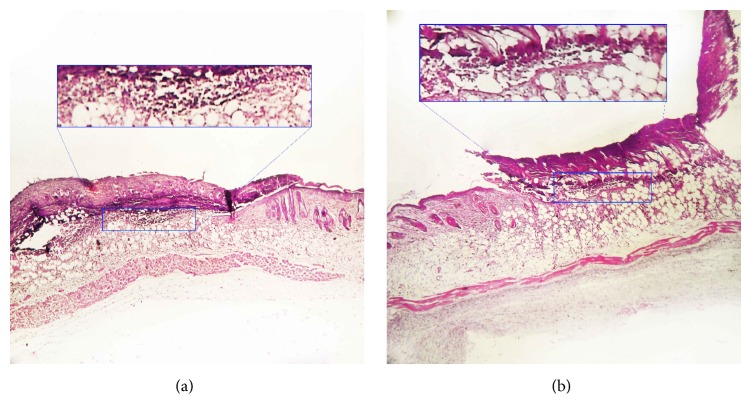
Photomicrographs of skin in Emu oil (a) and control (b) groups in the 4th day. Inflammatory cell density was higher in the Emu oil treated group in comparison with control group. Marked rectangle. Staining: Hematoxylin-Eosin, magnification: 40x.

On day 4, the fibroblast density and granulation tissue formation and keratosis on the edge of the wound in the Emu oil group were lower than those of the control group (Figures 1(a) and 1(b)), whereas inflammatory cell density was higher in the Emu oil group. No differences were observed in the other parameters (Table 1).

### 3.2. Microscopic Features of Wounds on Day 7

**Figure 2:**
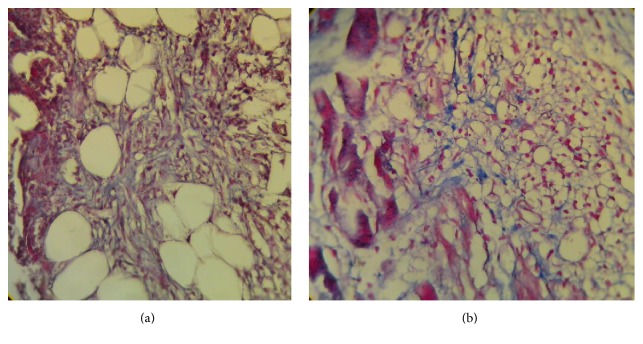
Photomicrographs of skin in emu oil (a) and control (b) groups in the 7th day. High active fibroblasts and collagen fibers in Emu oil treated group in comparison with the control group. Staining: Trichrome-Mallory, magnification: 400x.

On day 7, the inflammatory cell density, the PMNs, edema, the number of hair follicles on the edge of the wound and keratosis were higher in the Emu oil group than in the control group. The thickness of epidermis in the margin and the thickness of collagen fibers in the two groups were the same. The granulation tissue formation and the fibroblast density and activity were lower in the Emu oil group (Figures 2(a) and 2(b)) (Table 1).

### 3.3. Microscopic Features of Wounds on Day 10

On day 10, the inflammatory cell density, the PMNs, granulation tissue formation, edema, the number of hair follicles on the edge of the wound and keratosis, fibroblast density and activity, and collagen formation were higher in the Emu oil group (Table 1).

### 3.4. Microscopic Features of Wounds on Day 14

**Figure 3:**
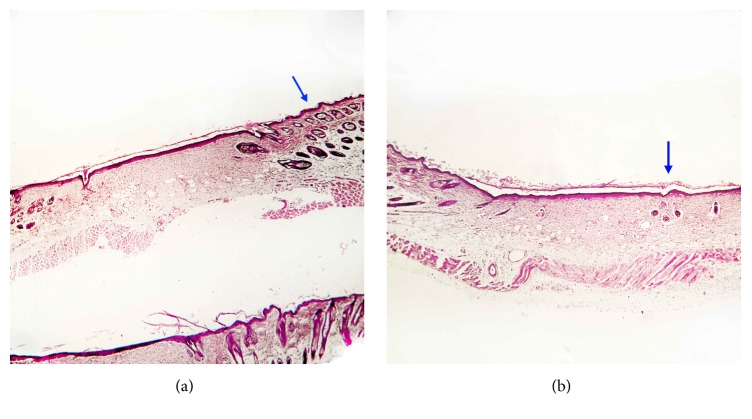
Photomicrographs of skin in emu oil (a) and control (b) groups in the 14th day. Thickness measure of epidermis in the margin of the wound and the number of hair follicles on the edge of the wound were higher in the Emu oil treated group in comparison with control group. Staining: Hematoxylin-Eosin, magnification: 40x.

On day 14, the inflammatory cell density, the PMNs, granulation tissue formation, edema, the thickness of epidermis in the margin of the wound, and the number of hair follicles on the edge of the wound were higher in the Emu oil group (Figures 3(a) and 3(b)).

The fibroblast density and activity and the keratosis in the edge of wound did not have any significant differences between two groups (Table 1).

The basal layer in both groups was completely destroyed by day 4. On day 7, it had begun to be reconstructed on the edges. On day 10, the edges had reached, and, on day 14, the reconstruction process was complete. Also, no hair follicles in the scars of both groups on each day of the experiment were observed.

## 4. Discussion

Emu oil has been used to enhance wound healing in complementary medicine for a long time. Today, there is evidence supporting the anti-inflammatory properties of the Emu oil, although a number of studies have claimed that the early application of Emu oil on burn wounds can elongate inflammatory phases and delay the healing process [14]. Similar results were obtained from this study.

On all days of this experiment, the inflammatory cell density was higher in the Emu oil groups in comparison with control groups. Also, considering the type of inflammatory cells, the polymorphonuclears were always dominant in the Emu oil groups. Edema was the same in both groups on day 4 (upmost degree). However, in the control group, the edema decreased more rapidly compared to Emu oil group in the following days. Politis and Dmytrowich showed that the immediate application of Emu oil on burn wounds delays the healing process, whereas its application after 48 hours enhanced the speed of the healing process twofold [14]. This result was consistent with our study; we applied Emu oil immediately after the burn and the inflammation and healing process were elongated. On the other hand, there are studies which show that Emu oil mitigates the inflammation process in the skin and also other organs [12, 13, 16]. López et al. reported that carotene-induced inflammation on mice oracles reduced significantly 6 hours after Emu oil application [12].

Snowden and Whitehouse revealed that the topical application of Emu oil can reduce tuberculosis-induced arthritis in rats' paws [13]. Another study carried out by Li et al. showed that Emu oil, in comparison to povidone Iodine and liquid paraffin, had better healing effects on burn wounds [16]. More detailed studies have shown that the anti-inflammatory effects of Emu oil are due to its ability to decrease some preinflammatory cytokines [17–19].

A possible reason for the difference in the inflammatory effects of Emu oil in the mentioned studies could be the use of enhancers along with the Emu oil, or other factors such as the purity and consistency [20].

Snowden and Whitehouse assessed the anti-inflammatory activity of five different preparations of Emu Oil varying in farm location, source of Emu adipose tissue (retroperitoneal or subcutaneous), rendering condition, and storage on the rat paws which topically received Emu oil following polyarthritis induction. They observed different anti-inflammatory effects of these compounds [13]. Beckerbauer et al. also demonstrated that diet composition of Emu can significantly influence the composition of Emu oil (especially fatty acids) and hence oil efficacy [10]. Although the mechanism of action of Emu oil and the nature of the active factors are still unknown, it has been suggested that n-3 and n-9 fatty acids in Emu oil may render inflammatory properties [21].

In present study, the density of the fibroblasts in the Emu oil group was lower in the 4th and 7th day and was higher in the 10th day of the experiment in comparison with control group (*P* < 0.05). On the other hand, the activity of fibroblasts nearly showed this similar pattern in the both of experimental groups. Granulation tissue formation in Emu oil group had delaying time until 7th day of experiment in comparison with control group but afterwards it proceeded with more speed compared to control group. This delaying time in proliferation phase of repairing process in Emu oil group may be related to the postponement that occurred in the inflammatory phase.

In this study, the relative frequency of keratinization on the surface of the wounds which was the same in both groups on day 4 was higher in the Emu oil group on other days, reaching its maximum on day 10. This shows that the topical application of Emu oil on burn wounds on the skin of Balb/c mice might improve the wound healing process by increasing the keratinization of the epidermis. This finding is in accordance with the Politis and Dmytrowich study that reported that Emu oil improved wound healing through the mechanism of enhanced keratinization [14]. In this study, the basal layer in both groups was completely destroyed on day 4, and there were attachments on the edges on day 7. On day 10, the basal layer was totally attached and was normal on day 14.

There were no hair follicles on the scar in both groups on different days of experiment. The effect of Emu oil on increasing the number of hair follicles was significant on the edge of the wound. The average number of hair follicles on the edge of the wound on day 4 was the same and around the normal rate (20/mm) in both groups, but, during the following days, increased significantly (*P* < 0.05) in the Emu oil group compared to the control group. (On days 7, 10, and 14, it was 22/mm, 25/mm and 28/mm, resp.)

While the wounds in the control group showed a decrease of hair follicles over time Holick removed the body hair of mice with the use of wax in a study in Boston University in 1998. Then, Emu oil was topically applied over an 18-day period. A tissue sample was collected in order to study the amount of hair follicles. Histological studies showed that the hair follicles in the Emu oil group were more and bigger compared to the control group [22]. Although this study did not show anything regarding an increase in the number of hair follicles per surface unit, we can deduce, based on its results and the results of this study, that the Emu oil has no effect on increasing the number of hair follicles in the scar (no hair can grow on the scar), but it can lead to an increase in the number of hair follicles on the edge of the wound, even more than the normal rate; and thus the scar would be smaller and less visible.

## 5. Conclusion

The findings indicate that although Emu oil delays the healing process at the inflammatory stages, it has a positive effect on wound healing especially keratinization of epidermis, and it also increases the number of hair follicles in the margins of the wound. Further studies are required to fully understand the molecular mechanism of repairing of Emu oil.

## Figures and Tables

**Table 1:** Microscopic features of wounds in Emu oil group and control group in different days.

	Day 4		Day 7		Day 10		Day 14	
	Case	Control	Case	Control	Case	Control	Case	Control
Inflammatory cell density (cell/HPF)	145 ± 56 Cell/HPF (++++)	98 ± 35 Cell/HPF (++++/+++)	91 ± 33 Cell/HPF (+++/+++)	72 ± 31 Cell/HPF (+++/++)	67 ± 28 Cell/HPF (+++/++)	29 ± 15 Cell/HPF (++/+)	21 ± 14 Cell/HPF (+)	12 ± 9 Cell/HPF (+/−)

Inflammatory cell type	100% PMN, no MN	98% PMN, 2% MN	93% PMN, 7% MN	70% PMN, 30% MN	80% PMN, 20% MN	5% PMN, 95% MN	30% PMN, 70% MN	No PMN, 100% MN

Granulation tissue formation	—	−/+	+	++	++	+/−	+/−	—

Edema	++	++	++/+	+	+	+/−	+/−	—

Fibroblast density	3 ± 2/HPF	5 ± 2/HPF	9 ± 4/HPF	13 ± 7/HPF	23 ± 12/HPF	18 ± 8/HPF	22 ± 10/HPF	20 ± 11/HPF

Fibroblast activity	—	—	−/+	+	++	+/++	++	++

Basal layer	Damaged	Damaged	Marked in margins	Marked in margins	Join completed	Join completed	Completed	Completed

Increased thickness of epidermis in the edge of wound	No epidermis in the edge	No epidermis in the edge	4 ± 1 layers' epidermis	4 ± 1 layers' epidermis	2 ± 1 layers' epidermis	2 ± 1 layers' epidermis	1 ± 1 layers' epidermis	No epidermis in the edge

Keratosis in the edge of wound	2 ± 1 layers	2 ± 1.5 layers	3 ± 1.5 layers	2 ± 1 layers	6 ± 2 layers	2 ± 1 layers	3 ± 1 layers	2 ± 1 layers

Number of hair follicles in the scar	12 ± 5 necrotic follicles/LPF	15 ± 6 necrotic follicles/LPF	0	0	0	0	0	0

Number of hair follicles in the margins	20 ± 2	20 ± 2.5	22 ± 3	18 ± 2.6	25 ± 3.4	15 ± 1.4	28 ± 2.6	12 ± 1.4

Microscope: Olympus SZX research microscope. LPF (Low Power Field) is 100x and HPF (High Power Field) is 400x. Diameter of objective lens of LPF is 1.02 mm and HPF is 0.51 mm.

## References

[1] Ghaderi R., Afshar M., Akhbarie H., Golalipour M. J. (2010). Comparison of the efficacy of honey and animal oil in accelerating healing of full thickness wound of mice skin. *International Journal of Morphology*.

[2] Peck M. D. (2011). Epidemiology of burns throughout the world. Part I: distribution and risk factors. *Burns*.

[3] Hosseinimehr S. J., Khorasani G., Azadbakht M., Zamani P., Ghasemi M., Ahmadi A. (2010). Effect of aloe cream versus silver sulfadiazine for healing burn wounds in rats. *Acta Dermatovenerologica Croatica*.

[4] Tarameshloo M., Norouzian M., Zarein-Dolab S., Dadpay M., Mohsenifar J., Gazor R. (2012). Aloe vera gel and thyroid hormone cream may improve wound healing in Wistar rats. *Anatomy & Cell Biology*.

[5] Upadhyay N. K., Kumar R., Mandotra S. K. (2009). Safety and healing efficacy of Sea buckthorn (*Hippophae rhamnoides* L.) seed oil on burn wounds in rats. *Food and Chemical Toxicology*.

[6] Khorasani G., Hosseinimehr S. J., Zamani P., Ghasemi M., Ahmadi A. (2008). The effect of saffron (*Crocus sativus*) extract for healing of second-degree burn wounds in rats. *Keio Journal of Medicine*.

[7] Gore M. A., Akolekar D. (2003). Evaluation of banana leaf dressing for partial thickness burn wounds. *Burns*.

[8] Ghaderi R., Afshar M. (2004). Topical application of honey for treatment of skin wound in mice. *Iranian Journal of Medical Sciences*.

[9] Whitehouse M. W., Turner A. G., Davis C. K. C., Roberts M. S. (1998). Emu oil(s): a source of non-toxic transdermal anti-inflammatory agents in aboriginal medicine. *Inflammopharmacology*.

[10] Beckerbauer L. M., Thiel-Cooper R., Ahn D. U., Sell J. L., Parrish F. C., Beitz D. C. (2001). Influence of two dietary fats on the composition of emu oil and meat. *Poultry Science*.

[11] Hilditch P. N., Williams T. P. (1964). *The Chemical Constitution of Natural Fats*.

[12] López A., Sims D. E., Ablett R. F. (1999). Effect of emu oil on auricular inflammation induced with croton oil in mice. *American Journal of Veterinary Research*.

[13] Snowden J. M., Whitehouse M. W. (1997). Anti-inflammatory activity of emu oils in rats. *Inflammopharmacology*.

[14] Politis M. J., Dmytrowich A. (1998). Promotion of second intention wound healing by emu oil lotion: comparative results with furasin, polysporin, and cortisone. *Plastic and Reconstructive Surgery*.

[15] Gong Z., Wang J., Fang X., Qiu X., Li Z., Yi C. (2005). Anti-inflammatory activity and healing-promoting effects of topical application of emu oil on wound in scalded rats. *Di Yi Jun Yi Da Xue Xue Bao*.

[16] Li Z.-Q., Wang J.-H., Ren J.-L., Yi Z.-H. (2004). Effects of topical emu oil on wound healing in scalded rats. *Di Yi Jun Yi Da Xue Xue Bao*.

[17] Balto K., Sasaki H., Stashenko P. (2001). Interleukin-6 deficiency increases inflammatory bone destruction. *Infection and Immunity*.

[18] Hou L., Sasakj H., Stashenko P. (2000). B-cell deficiency predisposes mice to disseminating anaerobic infections: Protection by passive antibody transfer. *Infection and Immunity*.

[19] Wang C. Y., Stashenko P. (1993). The role of interleukin-1 alpha in the pathogenesis of periapical bone destruction in a rat model system. *Oral Microbiology and Immunology*.

[20] Hashida M., Yamashita F., Smith E. W., Maibach M. I. (1995). Terpenes as penetration enhancers. *Percutaneous Penetration Enhancers*.

[21] Abimosleh S. M., Tran C. D., Howarth G. S. (2012). Emu oil: a novel therapeutic for disorders of the gastrointestinal tract?. *Journal of Gastroenterology and Hepatology*.

[22] Holick M. F. Use of emu oil for stimulating skin and hair growth.

